# Radiomics analysis based on multiparametric magnetic resonance imaging for differentiating early stage of cervical cancer

**DOI:** 10.3389/fmed.2024.1336640

**Published:** 2024-02-02

**Authors:** Feng Wu, Rui Zhang, Feng Li, Xiaomin Qin, Hui Xing, Huabing Lv, Lin Li, Tao Ai

**Affiliations:** ^1^Department of Radiology, Xiangyang Central Hospital, Affiliated Hospital of Hubei University of Arts and Science, Xiangyang, China; ^2^Department of Obstetrics and Gynaecology, Xiangyang Central Hospital, Affiliated Hospital of Hubei University of Arts and Science Xiangyang, China; ^3^Department of Radiology, Tongji Hospital, Tongji Medical College, Huazhong University of Science and Technology, Wuhan, China

**Keywords:** Radiomics, magnetic resonance imaging, cervical cancer, treatment, multiparametric

## Abstract

**Objective:**

To investigate the performance of multiparametric magnetic resonance imaging (MRI)—based radiomics models in differentiating early stage of cervical cancer (Stage I-IIa vs. IIb-IV).

**Methods:**

One hundred patients with cervical cancer who underwent preoperative MRI between June 2020 and March 2022 were retrospectively enrolled. Training (*n* = 70) and testing cohorts (*n* = 30) were assigned by stratified random sampling. The clinical and pathological features, including age, histological subtypes, tumor grades, and node status, were compared between the two cohorts by *t*-test or chi-square test. Radiomics features were extracted from each volume of interest (VOI) on T2-weighted images (T2WI) and apparent diffusion coefficient (ADC) maps. The data balance of the training cohort was resampled by synthesizing minority oversampling techniques. Subsequently, the adiomics signatures were constructed by the least absolute shrinkage and selection operator algorithm and minimum-redundancy maximum-relevance with 10-fold cross-validation. Logistic regression was applied to predict the cervical cancer stages (low [I-IIa]) and (high [IIb–IV] FIGO stages). The receiver operating characteristic curve (area under the curve [AUC]) and decision curve analysis were used to assess the performance of the radiomics model.

**Results:**

The characteristics of age, histological subtypes, tumor grades, and node status were not significantly different between the low [I-IIa] and high [IIb–IV] FIGO stages (*p* > 0.05 for both the training and test cohorts). Three models based on T2WI, ADC maps, and the combined were developed based on six radiomics features from T2WI and three radiomics features from ADC maps, with AUCs of 0.855 (95% confidence interval [CI], 0.777–0.934) and 0.823 (95% CI, 0.727–0.919), 0.861 (95% CI, 0.785–0.936) and 0.81 (95% CI, 0.701–0.918), 0.934 (95% CI, 0.884–0.984) and 0.902 (95% CI, 0.832–0.972) in the training and test cohorts.

**Conclusion:**

The radiomics models combined T2W and ADC maps had good predictive performance in differentiating the early stage from locally advanced cervical cancer.

## 1 Introduction

Cervical cancer (CC) is one of the most common malignant among women worldwide and also the second leading cause of cancer deaths among women in China (1–3). Although evidence shows that the incidence of CC in developed countries is declining (3, 4), the age-standardized morbidity and mortality of CC in China have shown a significant upward trend (3). In China, the incidence rate of CC has increased from 10 to 40% over the past 30 years (3).

International Federation of Obstetrics and Gynecology (FIGO) staging of CC has always been the staging system commonly used in clinical diagnosis and treatment. CC is primarily managed by surgical treatment or radiotherapy, with chemotherapy as a valuable adjunct. Surgery is the first choice for treating stage IA1, IA2, IB1, IB2, and IIA1 lesions (5). Concurrent chemoradiation is the standard treatment for stages IB3, IIA2, III, and IV diseases. A radical trachelectomy can be performed for young women interested in preserving their fertility, and it is suitable for stage IA2–IB1 tumors with a maximum diameter of no more than 2 cm (5). Given the excellent prognosis of early-stage CC and that as many as 40% of the women affected by these tumors are of reproductive age, fertility-sparing surgery has become a priority (6).

Magnetic resonance imaging (MRI) can noninvasively assess tumor size and the extent of invasion owing to its merits of multiparametric and multidirectional imaging with high soft tissue resolution (7, 8). Therefore, imaging is complementary to clinical assessment with MRI, which is accepted as the optimal modality for staging CC. Important information about the morphology and extent of interstitial invasion of CC can be obtained from T2-weighted (T2W) images (9). The apparent diffusion coefficient (ADC) maps provide information about water fluidity and tissue cell structure to characterize cancer quantitatively (10).

The heterogeneity of CC is inconsistent among different FIGO stages, histological subtypes, and tumor grades (9). It is an essential factor that can predict tumor aggressiveness and could also be reflected in MRI. However, these heterogeneities may be considered similar just by visual assessment of MRI with the radiologist’s naked eye. Radiomics is an evolving field that involves extracting many quantitative features from images, such as MRI, computed tomography, and ultrasound, and using a high-throughput process that effectively transforms images into quantitative data to provide more valuable information (11). A series of quantitative features that have been generated can be further used to measure intra-tumor heterogeneity. Currently, several recent studies have described the use of radiomics in CC, mostly on clinicopathological characteristics (9, 12), parametrial invasion (13), pelvic lymph node metastases (14, 15), and predictive performance (16, 17). However, few studies have assessed the performance of radiomics in predicting the stage of cervical cancer, which essentially influences treatment decision-making in the clinical setting. Thus, this study aimed to investigate the predictive performance of multiparametric MRI-based radiomics models in differentiating the low (I-IIa) and high (IIb–IV) FIGO stages of cervical cancer.

## 2 Materials and methods

### 2.1 Patients enrollment

**Figure 1:**
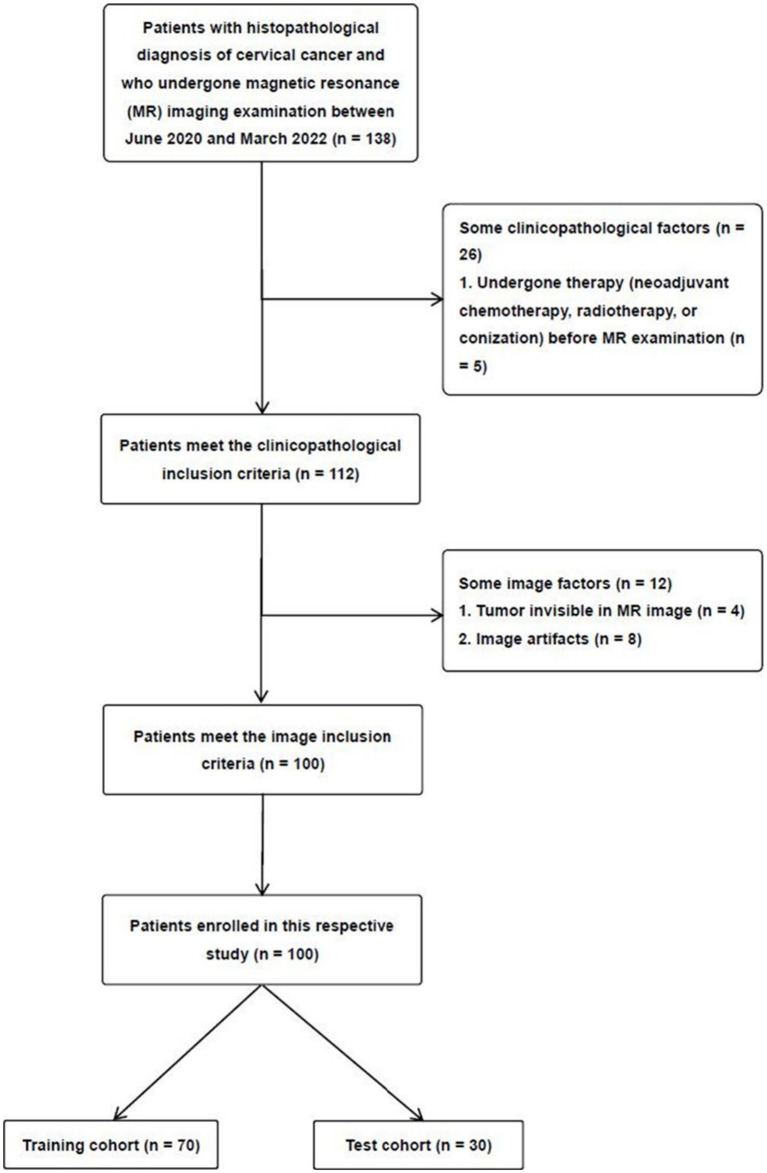
Schematic diagram for patient selection.

This study was approved by the institutional ethics review board of our hospital (approval no. 2022–027), and the informed consent requirement was waived due to the retrospective study. The patients were enrolled through the following inclusion criteria: (a) patients with histologically confirmed CC; (b) patients who have not undergone therapy (neoadjuvant chemotherapy, radiotherapy, or conization) before MRI examination; (c) patients undergoing T2-weighted imaging (T2WI) with fat suppression, and DWI with ADC maps; and (d) classify the cases based on the 2018 FIGO system. The exclusion criteria were as follows: (a) patients with tumors that were too small for the region of interest (ROI) to be accurately drawn, (b) patients with poor MRI image quality resulting from artifacts, (c) patients with incomplete clinicopathological data, and (d) patients with rare histological subtypes. Between June 2020 and March 2022, 100 patients with CC participated in our study. According to the proportion of 7:3, the 100 patients were randomly divided into two independent cohorts, a training and a test cohort (Figure 1).

All patients’ clinical and pathologic features, including age, treatment, FIGO stage, pathological information, and serum squamous cell carcinoma antigen (SCC-Ag) levels before treatment, were derived from the medical records. The treatments were divided into surgical and non-surgical treatments. FIGO stages were dichotomized into low (I-IIa) and high (IIb–IV) FIGO stages. The assessed pathological information comprised histological subtypes, tumor grades, invasion depth, and lymphovascular space invasion (LVSI) according to the World Health Organization Classification of Tumors of Female Reproductive Organs. There are two histological subtypes of squamous cell carcinoma (SCC) and adenocarcinoma (ACA). Tumor grades were divided into three groups: well (G1), moderately (G2), and poorly differentiated (G3). Invasion depth was classified into inner, middle, and outer layers. After reviewing the MRI of all patients, the node status was recorded by two radiologists with five and more than ten years of experience in gynecological cancer diagnosis, respectively. Any disagreements were resolved by discussion and consensus. Nodal status was based on T2WI. The positive lymph node was defined as the short axis of the lymph node >10 mm, spiculated or lobulated margin, or internal necrosis (9).

### 2.2 MRI acquisition

All preoperative MR examinations were performed with a 3.0 T platform with respiratory gating technology and an eight-channel phased array body coil (Siemens Medical Solutions, Verio 3.0, Germany). All recruited patients underwent T2W fat-suppressed and diffusion-weighted imaging (DWI) sequences acquired before surgery or chemoradiation. The ADC was calculated according to the traditional single exponential model, and patients were advised to fast for 5–6 h before examination (9). Conventional MRI comprised oblique axial T2W images (echo time [TE], 82 ms; repetition time [TR], 3,800 ms; gap, 2 mm; slice thickness, 5 mm; field of view [FOV], 320 × 320 mm) with fat suppression and transverse DWI (TE, 52 ms; TR, 3900 ms; gap, 2 mm; slice thickness, 5 mm; FOV, 320 × 256 mm; and b values, 50 and 800 s/mm^2^).

### 2.3 Image segmentation and radiomics feature extraction

The solid lesions’ three-dimensional volumes of interest (VOIs) were manually segmented using ITK-SNAP (version 3.8.0), a free and open-source software. A junior radiologist (with five years of experience in diagnosing gynecologic cancer) manually delineated the low-signal rim of the tumor from adjacent normal tissue, excluding high-signal areas within the lesion, on high-spatial-resolution axial T2W images. The VOI segmentation was performed on DWI with a b value of 800 s/mm^2^ and then mapped into the ADC image. All segmented VOIs were confirmed and corrected by a senior radiologist (with >10 years’ experience in gynecological tumor diagnosis). The radiologists were blinded to the clinicopathological results. Another junior radiologist (three years of experience in diagnosing gynecological diseases) independently performed manual segmentation of these lesions to analyze interobserver reproducibility. The radiologists performed manual segmentation blinded to diagnostic information such as clinical and histopathology.

Python (version 3.7.5) with the PyRadiomics package (https://github.com/AIM-Harvard/pyradiomics.git, version 3.0.1) extracted radiomics features from T2W and ADC images. All radiomics features were extracted from the original image and wavelet-filtered image, which could be divided into three groups: 18 first-order statistics, 14 shape features, and 75 textural features. The feature extraction method is provided on an official website.^1^ Before radiomics feature extraction, all T2W and ADC maps were normalized using the z-score method and voxel size resampling by 1 × 1 × 1 mm. Finally, 851 features are extracted in each VOI of the T2W and ADC images, respectively.

### 2.4 Feature selection and radiomics model building

The interobserver reproducibility of each radiomics feature was assessed using the interclass correlation coefficient (ICC). The ICC > 0.80 was considered excellent and included in subsequent analyses. The synthetic minority oversampling technique (SMOTE) method dealt with the balance of balanced categories in the training cohort to prevent bias in the construction of the predictive model because the sample size of the high FIGO stage was 33.3% less than the sample size of the low FIGO stage. The training dataset used for model building analysis was the training dataset after SMOTE processing (training-SMOTE cohort). The low/high FIGO stage of CC patients were 1:1 (42 low FIGO stage patients and 42 high FIGO stage patients) in the SMOTE-training cohort.

To reduce redundancy, spearman correlation analysis was used to eliminate features with a Spearman correlation coefficient > 0.9. The top 10 features with low redundancy and high correlation with CC were selected for the following analysis using the minimum redundancy maximum correlation (mRMR) algorithm. Then, the least absolute shrinkage and selection operator (LASSO) method was used to screen the radiomics features that helped predict the CC therapy method in the training cohort with SMOTE. A total of 10 cross-validation methods were used to identify the model’s generalization performance in the LASSO method. The single radiomics model using T2W images (T2 model) and ADC maps (ADC model) was weighted using coefficients of selected features with optimal adjustment weights in LASSO logistic regression. The combined model was developed based on multivariate analysis’s T2 and ADC models.

### 2.5 Statistical analyses

We conducted differences analysis of the characteristics of patients between the training and test cohorts, using the chi-square test for categorical variables and Student’s *t*-test for continuous variables. The interobserver reproducibility of the radiomics features evaluating the interobserver agreement among radiologists was accessed using the ICC, with a coefficient greater than 0.8, indicating good reproducibility. Receiver operating characteristic (ROC) curve analysis was used to assess the diagnostic performance of the radiomics models for predicting CC. The optimal cutoff value for predictive diagnosis for radiomics models was determined by maximizing the Youden index in the training cohort with SMOTE. The areas under the ROC (AUC), accuracies, specificities, sensitivities, negative predictive values, and positive predictive values were used to quantify the diagnostic performance of the radiomics models. Decision curve analysis (DCA) was used to assess the clinical usefulness of the models. This study’s statistical analysis was performed using R (version 3.6.1, https://www.r-project.org). *p* < 0.05 (two-tailed) was considered to be statistically significant.

## 3 Results

### 3.1 Clinical characteristics

The clinical characteristics of the patients are summarized in Table 1. In the invasion depth, LVSI, and tumor grade groups, the pathological information of some patients not undergoing surgery (such as those receiving chemotherapy) was missing. In 100 patients with CC (mean age, 53.48 ± 10.58 years), 90 and 10 had SCC and ACA, respectively. All patients in the training and test cohorts were further divided into the low (*n* = 61) and high (*n* = 39) FIGO stage cohorts. The rates of low FIGO stages in the training and test cohorts remained balanced (0.600 and 0.633, respectively, *p* = 0.374). The clinical and pathologic characteristics, including age and histological subtype, were not significantly different between the two cohorts (*p* > 0.05). Furthermore, the MR-reported nodal status was not significantly different between the low and high FIGO stage cohorts (*p* = 0.385).

**Table 1:** Clinical characteristics of the patients with CC in the training and test cohorts.

Characteristics	Overall	Training cohort	Test cohort	*p*	SMD	Missing
	100	70	30			
Age (mean [SD])	53.480 (10.580)	55.260 (10.610)	50.980 (11.250)	0.380	0.070	0
Nodal status				0.385	0.255	0
Positive (%)	24 (24.000)	19 (27.143)	5 (16.667)			
Negative (%)	76 (76.000)	51 (72.857)	25 (83.333)			
Before SCC (median [IQR])	1.960 (0.880–5.850)	2.040 (0.850–5.910)	3.080 (1.270–4.890)	0.958	0.041	0
Neoadjuvant chemotherapy				0.269	0.316	0
Positive (%)	22 (22.000)	18 (25.714)	4 (13.333)			
Negative (%)	78 (78.000)	52 (74.286)	26 (86.667)			
Surgery				0.374	0.243	0
Positive (%)	68 (68.000)	50 (71.429)	18 (60.000)			
Negative (%)	32 (32.000)	20 (28.571)	12 (40.000)			
Histological subtype				0.716	0.153	0
Adenocarcinoma (%)	10 (10.000)	6 (8.571)	4 (13.333)			
Squamous cell carcinoma (%)	90(90.000)	64(91.429)	26 (86.667)			
Tumor grades (%)				0.393	0.322	18
G1	6 (6.000)	3 (4.286)	3 (10.000)			
G2	15 (15.000)	12 (17.143)	3 (10.00)			
G3	61 (61.00)	43 (61.428)	18 (60.000)			
Depth (%)				0.801	0.164	22
Inner	23 (23.000)	15 (21.428)	8 (26.667)			
Middle	15 (15.000)	11 (15.714)	4 (13.333)			
Outer	40 (40.000)	25 (35.714)	15 (50.000)			
LVSI				0.954	0.076	22
Positive (%)	42 (42.000)	29 (41.428)	13 (43.333)			
Negative (%)	36 (36.000)	26 (37.143)	10 (33.333)			
FIGO stage (%)				0.337	0.502	0
I	42 (42.000)	30 (42.857)	12 (40.000)			
IIA	19 (19.000)	12 (17.142)	7 (23.333)			
IIB	8 (8.000)	4 (5.714)	4 (13.333)			
III	27 (27.000)	20 (28.571)	7 (23.333)			
IV	4 (4.000)	4 (5.714)	0 (0.000)			
FIGO group				0.374	0.056	
Low FIGO (I-IIa)	61 (61.000)	42 (60.000)	19 (63.333)			
High FIGO (IIb-IV)	39 (39.000)	28 (40.000)	11 (36.667)			

SD, standard deviation; SMD, standardized mean difference; IQR, interquartile range; LVSI, lymphovascular space invasion; FIGO, international federation of obstetrics and gynecology.

### 3.2 Radiomics model construction

**Figure 2:**
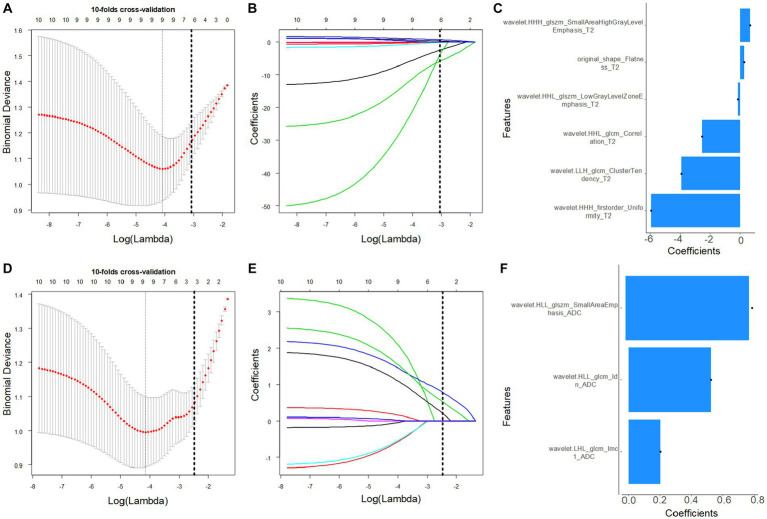
Magnetic resonance-based radiomics feature selection using the least absolute shrinkage and selection operator (LASSO) method in the training cohort. **(A,D)** The optimal penalty coefficient lambda (λ) for the feature of the T2W **(A)** and apparent diffusion coefficient (ADC) **(D)** images was obtained based on 10-fold cross-validation. **(B,E)** Changes in the corresponding coefficients of T2W and ADC image features during Lasso analysis. The vertical dashed line represents the optimal λ, corresponding to six (T2) and three (ADC) nonzero feature coefficients.

The low/high FIGO stage cohort in the training cohort was converted from 42/28 to 42/42 using the SMOTE method. In total, 221 features were screened from each VOI in a T2W image, and the features were reduced to six CC-related features after the application of the mRMR and LASSO algorithms in the training-SMOTE cohort (Figures 2A–C). Similarly, the 230 ADC radiomics features were reduced to three imaging biomarkers after applying the mRMR and LASSO algorithms in the training-SMOTE cohort (Figures 2D–F). The ICC ranges for T2W and ADC image radiomics features were 0.34–0.99 and 0.21–0.99, respectively. The T2 and ADC model calculation formulae were as follows:

T2 radiomics signature =

−1.624 – 5.923 × wavelet.HHH_firstorder_Uniformity

−3.922 × wavelet.LLH_glcm_ClusterTendency

−2.531 × wavelet.HHL_glcm_Correlation

−0.163 × wavelet.HHL_glszm_LowGrayLevelZoneEmphasis

+0.271 × original_shape_Flatness

+0.660 × wavelet.HHH_glszm_SmallAreaHighGrayLevelEmphasis

ADC radiomics signature =

0.431 + 0.431 × wavelet.LHL_glcm_Imc1

+0.523 × wavelet.HLL_glcm_Idn

+0.782 × wavelet.HLL_glszm_SmallAreaEmphasis

### 3.3 Radiomics model performance

**Figure 3:**
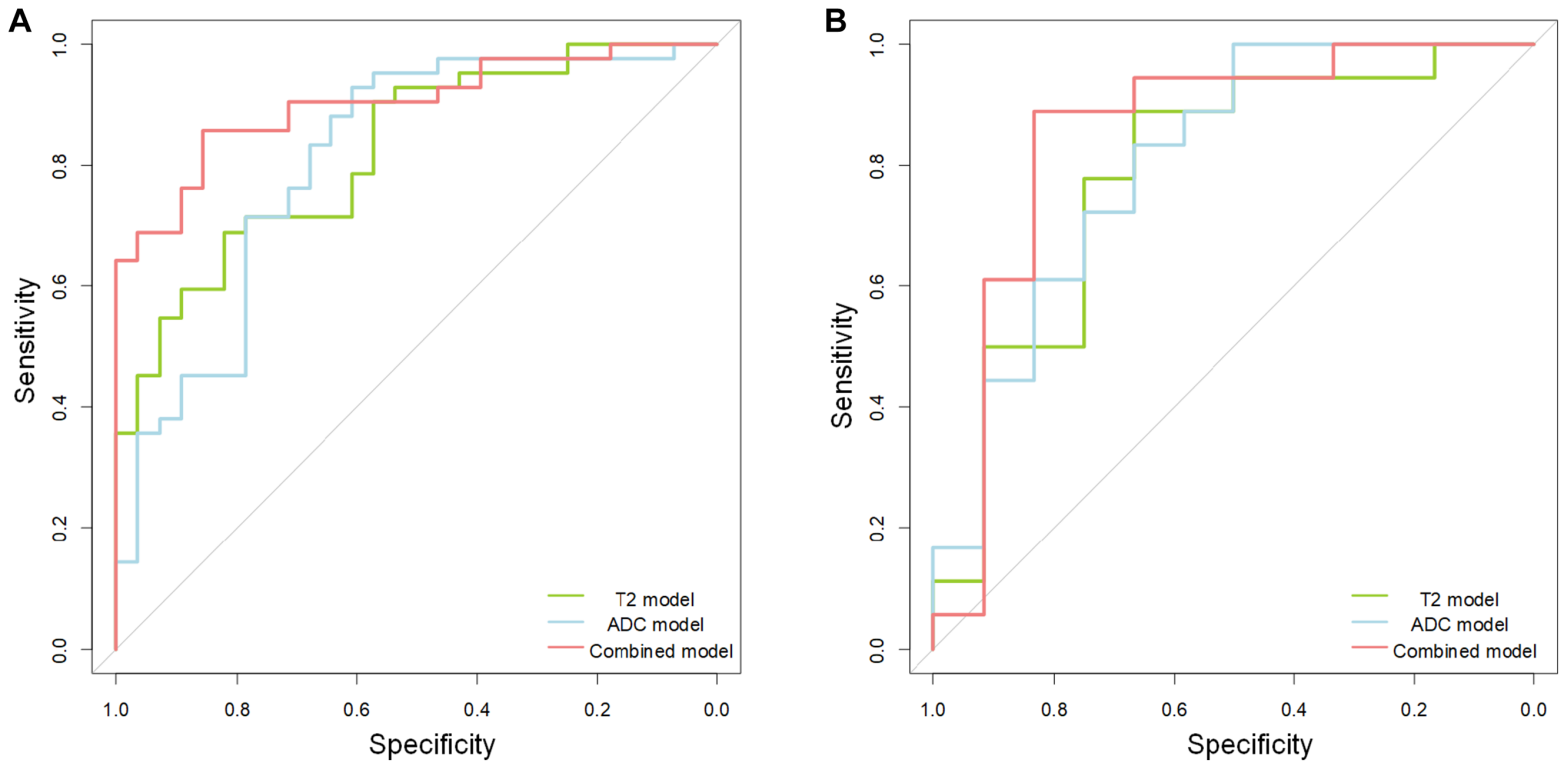
The receiver operating characteristic (ROC) curves of the T2 (green line), apparent diffusion coefficient (ADC) (blue line), and combined (red line) model. The receiver operating characteristic curve shows the combined model is better than the separate T2 and ADC model in the training **(A)** and test **(B)** cohorts.

**Figure 4:**
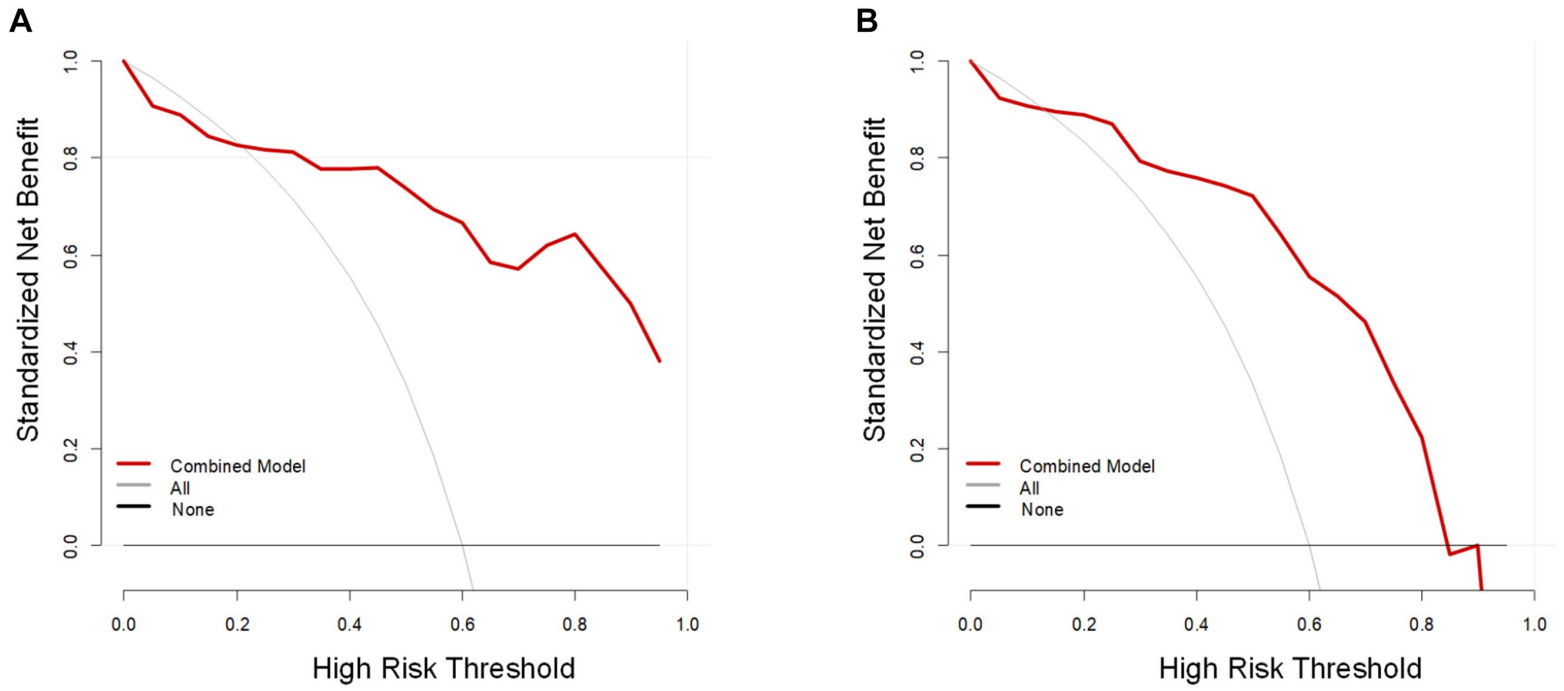
The decision curve analysis (DCA) of the combined model (red line). The vertical and horizontal axes represent net benefit and threshold probability, respectively. The DCA revealed that the combined model could provide benefits in the training cohort **(A)**, with the threshold probability between 0.200 and 1, and in the test cohort **(B)**, with the threshold probability between 0.150 and 0.850.

In the training cohort, the AUCs of the T2W, ADC, and combined models of predicting CC were 0.823 (95% CI, 0.727–0.919), 0.810 (95%CI, 0.701–0.918), and 0.902 (95% CI, 0.832–0.972), respectively. In the test cohort, the AUCs were 0.829 (95% CI, 0.658–0.999), 0.773 (95% CI, 0.578–0.969), and 0.856 (95% CI, 0.707–1.000). The sensitivities of the three models for predicting CC were 0.929 and 0.889, 0.762 and 0.722, 0.857 and 0.833 in the training and test cohorts. In the training and test cohorts, the specificities of the three models were 0.536 and 0.583, 0.714 and 0.667, and 0.857 and 0.833. The accuracies were 0.771 and 0.767, 0.743 and 0.700, and 0.857 and 0.833 in the training and test cohorts. The AUCs, accuracies, sensitivities, and specificities of the three models are shown in Table 2. Figure 3 shows the ROC curves of the three models. The results showed that the combined model had better diagnostic and predictive performance than the T2W and ADC models alone. The DCA was applied to evaluate the clinical usefulness, showing that the combined model could provide benefits in the training cohort (Figure 4A), with the threshold probability greater than 0.200, and in the test cohort (Figure 4B), with the threshold probability between 0.150 and 0.850.

**Table 2:** Performance of the sequences models.

		AUC	SEN	SPE	ACC	NPV	PPV
T2 model	Training cohort	0.823 (0.727–0.919)	0.929 (0.833–1.000)	0.536 (0.357–0.714)	0.771 (0.656–0.863)	0.833 (0.661–1.000)	0.750 (0.632–0.868)
	Test cohort	0.787 (0.604–0.969)	0.889 (0.722–1.000)	0.583 (0.333–0.833)	0.767 (0.577–0.901)	0.778 (0.506–1.000)	0.761 (0.579–0.944)
ADC model	Training cohort	0.810 (0.701–0.918)	0.762 (0.619–0.881)	0.714 (0.536–0.857)	0.743 (0.624–0.840)	0.667 (0.624–0.840)	0.800 (0.676–0.924)
	Test cohort	0.806 (0.634–0.977)	0.722 (0.500–0.889)	0.667 (0.417–0.917)	0.700 (0.506–0.853)	0.615 (0.807–0.993)	0.764 (0.563–0.996)
Combined model	Training cohort	0.902 (0.832–0.972)	0.857 (0.738–0.952)	0.857 (0.714–0.964)	0.857 (0.753–0.929)	0.800 (0.657–0.943)	0.900 (0.807–0.993)
	Test cohort	0.852 (0.683–1.000)	0.833 (0.667–1.000)	0.833 (0.583–1.000)	0.833 (0.653–0.944)	0.769 (0.540–0.998)	0.882 (0.729–1.000)

AUC, area under the curve; SEN, sensitivity; SPE, specificity; ACC, accuracy; PPV, positive predictive value; NPV, negative predictive value.

## 4 Discussion

In our study, we successfully constructed a radiomics model for the preoperative prediction of CC therapy (surgical and non-surgical treatment). The radiomics model incorporated the ADC map and T2W radiomics signature, and the results were validated in the test cohort. The established radiomics model demonstrated good discrimination and predictive power in both cohorts.

Treatment options for early-, intermediate-, and advanced-stage CC vary widely based on the latest FIGO staging system from 2018. Despite technological advancements in imaging-based diagnostics, many studies have shown that the morphological evaluation of MR images involves many subjective viewpoints. Many patients are misclassified, such as evaluating lymph node metastasis and parametrial invasion (18, 19).

Multidimensional characterization of radiomics features and quantification of detailed information in tumor images can reflect heterogeneity among different tumors. Therefore, preoperative radiomics signatures can provide a more objective and accurate assessment. Preoperative MR imaging can improve clinical staging accuracy by assessing the tumor’s location and size, parametrial invasion, and lymph node metastasis to select more appropriate treatment plans. Previous studies involving the radiomics method have noted their predictive value for diagnosing tumors and therapeutic effects accurately. Some of these studies have shown that the performance of radiomics models can be improved by using the high-throughput features of multiparametric images of tumor lesions (20, 21). Therefore, in our study, we extracted and screened some radiomics features from T2WI and ADC maps and finally obtained radiomics signatures based on T2WI and ADC. The results demonstrated that radiomics features from T2WI and ADC have roughly similar discrimination performance for therapy method prediction.

The T2WI-ADC-combined radiomics features contained more wavelet filtered features, most likely because the filter could map the image to several transform domains and better conveyed the tumor’s biological information (22). The wavelet transform can also gradually convert image information into low- and high-frequency information, which improves local features, increases information content in tumor images, and provides more information about the biological behaviors and heterogeneity of different tumors at multiple scales (22, 23). There was an original feature named original_shape_Flatness in the T2WI radiomics features. The lesions in the high FIGO stage cohort were more irregular in shape because of the larger size of the tumor, deep stromal invasion, or parametrial invasion than those in the low FIGO stage cohort (24). Lee, in a review, stated that the 2018 revision of FIGO requires a more accurate description of the size of primary tumors and should be measured using MRI, especially for cervical resection plans (25).

DWI describes water mobility within the lesion tissue and enables quantitative evaluation of the diffusion properties of diseased tissues according to the calculated ADC (10). This quantitative parameter has been used in many studies to characterize tumors or assess their response to treatment (24, 26). Several studies have found that in CC, the minimum ADC values of tumors have been related to SCC, tumor grades, parametrial invasion, and poor survival rate. Furthermore, the changes in ADC values of lesions during radiotherapy and chemotherapy are also associated with the treatment response of tumors (10, 24, 27, 28). Haldorsen et al. considered that the ADC value of the tumor provides additional information about the microstructure of the tumor that may be relevant for staging and prediction of CC (24).

T2WI can provide detailed morphological features of CC in patients, and the features extracted from T2WI in this study have high sensitivity and low specificity. The low specificity of T2WI may be due to high-signal edema or inflammation within the paracervical fascia, which is indistinguishable from high-signal tumors (29). However, a combination of T2 and ADC prediction models can solve this problem. Many previous studies have found that radiomics features extracted from T2WI can help predict cervical lymph node metastasis and parametrial infiltration (9, 16, 21).

In the 2018 edition of the FIGO staging system, preoperative MR lymph node status is directly involved in IIIC staging (5). We used this as an important independent factor in the study; the results did not perform relatively well. Previous studies have used different tumor diameters to forecast the risk of parametrial invasion in patients with early-stage CC, and the measurement and selecting standards for tumor diameter have also varied from the different studies (19, 30). Some studies have found no direct correlation between tumor diameter and lymph node metastasis (21). Prediction of a therapeutic method relying on tumor diameter might not apply in clinical settings. Previous studies have shown that patient age is also an important independent factor for para-uterine invasion and lymph node metastasis prediction of CC (31, 32); however, the results did not perform relatively well in predicting the CC therapy method in our study. Gravdal et al. reported that the incidence of CC in women aged <30 years has increased in European countries over the past 20 years, but overall, the cancer does not tend to be more advanced when detected (33). The same study from the UK concluded that CC in younger women (aged 20–24 years) tended to be more advanced than in older women and is often a rarer histological type (34). In our study, there were only four patients aged <30 years with pathology of SCC, and statistical differences may not have been noted. SCC-Ag is currently the most widely used biomarker for diagnosing and estimating the effect of chemotherapy in patients with CC (21, 32). Shou et al. found that the serum SCC-Ag level was statistically associated with advanced FIGO stage (35). However, some studies have shown no relationship between SCC-Ag level and clinical stage (36). We also included preoperative SCC-Ag levels as a clinical factor, and there was no correlation between SCC-Ag levels and prediction of the CC therapy method. However, further studies with larger sample sizes are warranted.

Our study has some limitations. First, MRI acquisition and segmentation were independently obtained by two radiologists using a consensus, and further studies are needed to validate inter- and intra-observer repeatability. Second, in our study, radiomics features extracted from T2W images had low specificity, and further research with larger sample sizes or wider range of clinical and imaging features are required. Furthermore, all subjects in our study had ACAs and squamous carcinomas. Different histological subtypes of CC should be thoroughly studied in the future.

In conclusion, radiomics models were constructed from the ADC maps and T2WI, which were robust in differentiating the low (I-IIa) and high (IIb–IV) FIGO stages of cervical cancer, which may be valuable for the therapy decision-making in cervical cancer. The results also suggest that the combination model based on T2WI and ADC maps had the best performance in predicting the CC stage.

## Data availability statement

The raw data supporting the conclusions of this article will be made available by the authors, without undue reservation.

## Ethics statement

This study was approved by the institutional ethics review board of our hospital (approval no. 2022-027), and the informed consent requirement was waived due to the retrospective study.

## Author contributions

FW: Conceptualization, Formal analysis, Investigation, Methodology, Writing – original draft. RZ: Data curation, Writing – original draft. FL: Validation, Writing – original draft. XQ: Methodology, Supervision, Validation, Writing – original draft. HX: Methodology, Supervision, Writing – review & editing. HL: Data curation, Writing – review & editing. LL: Formal analysis, Investigation, Writing – review & editing. TA: Writing – original draft, Writing – review & editing.

## Footnotes

^1^ https://pyradiomics.readthedocs.io/en/latest
